# Supplementary material for: Edge Effect in Electronic and Transport Properties of 1D Fluorinated Graphene Materials
Source: Nanomaterials (Basel). 2021 Dec 30;12(1):125. doi: 10.3390/nano12010125 (PMC8746569; doi:10.3390/nano12010125)
Supplement: Supplementary file 1 [file nanomaterials-12-00125-s001.zip › nanomaterials-1510604-supplementary.pdf]

Supporting Information: Edge Effect in Electronic and Transport Properties of 1D Fluorinated Graphene Materials

Jingjing Shao* and Beate Paulus

Institut für Chemie und Biochemie, Freie Universität Berlin, Arnimallee 22, 14195 Berlin, Germany

E-mail: jingjingshao@zedat.fu-berlin.de

Contents

1. **F-6ZGNRs Transport Model Convergence** **S-2**

2. **Band Structures and PDOS of Partially Fluorinated F-6ZGNRs** **S-4**

3. **Fluorinated F-8ZGNRs and F-12ZGNRs Structures** **S-5**

4. **Total Current-Bias Voltage Dependence of Odd Number Width of F-XZs** **S-8**

5. **Local Energy Spectra of Considered Structures** **S-9**

1. F-6ZGNRs Transport Model Convergence

1.1 Local energy spectra of F-6ZGNRs related to convergence of the transport models.

Local energy spectra are calculated via the secular equation $H_iU_i = S_iU_iE_i$ for each diagonal block of the matrix for i $= Electrodes(L), Scatteringregion, Electrodes(R)$, individually. All matrices of each segment are taken at the Γ point.

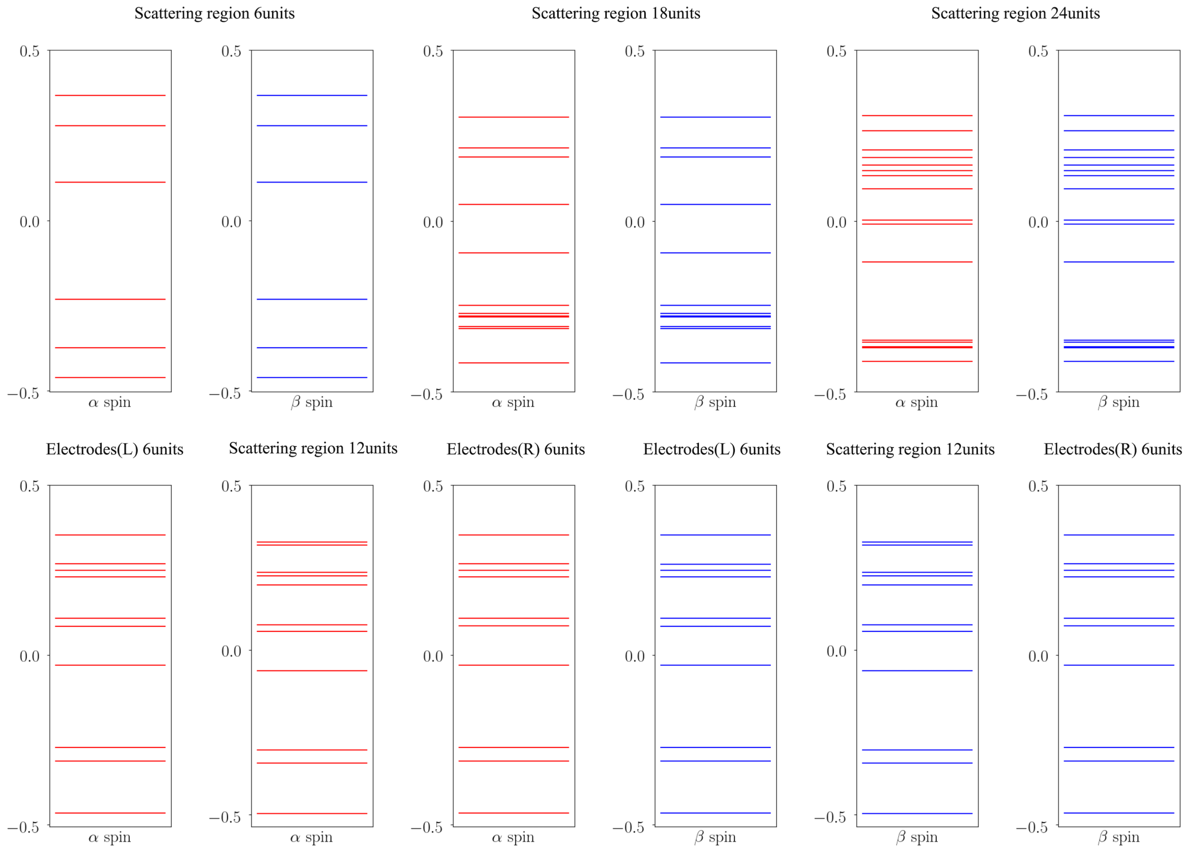

Figure S1: Local energy spectra of the different length of the scattering region and the electrodes (left and right with 6units each) of the F-6ZGNRs transport model. The α spin channel (red) is shown in the upper panel and the β spin channel (blue) is shown in the bottom. The upper panels show the scattering region with different length: 6, 18 and 24 units. The lower panel shows the converged scattering region with 12 units and the electrodes.

1.2 Transmission function of F-6ZGNRs with different η values.

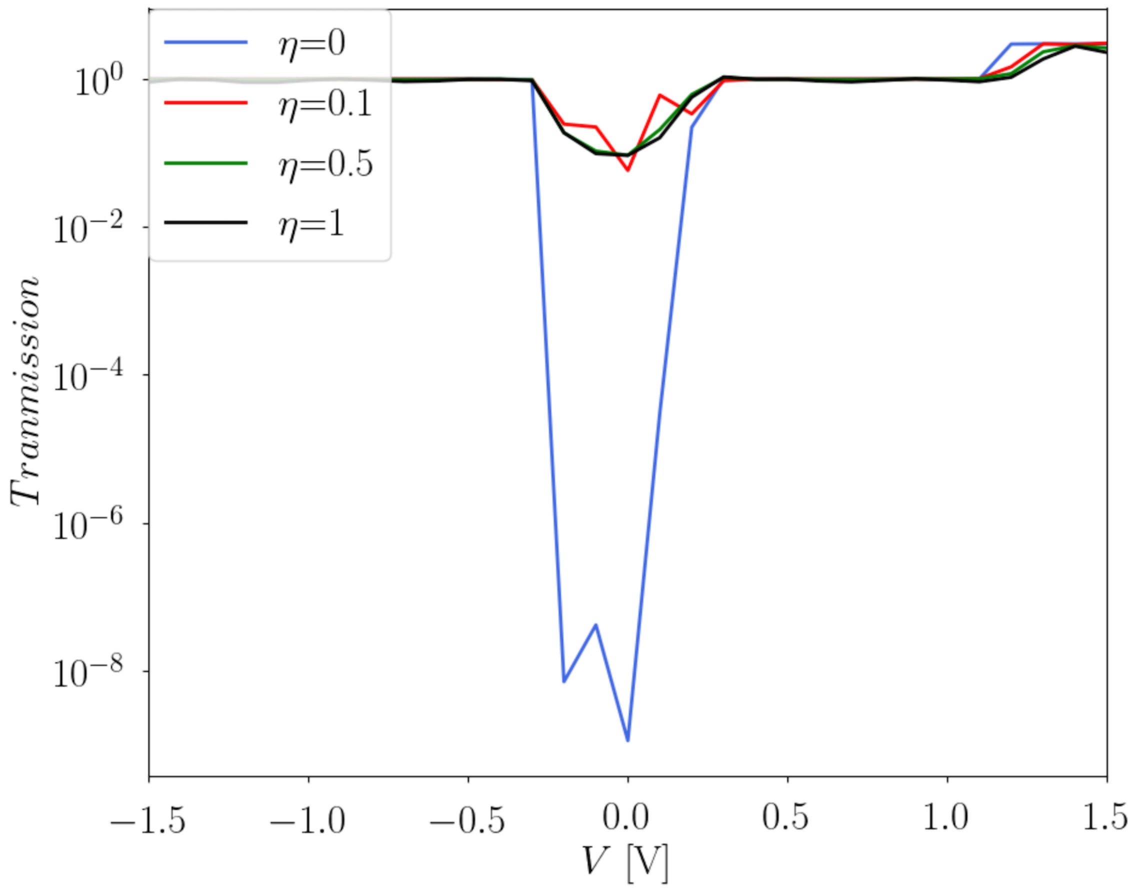

Figure S2: Transmission function of the scattering region of the F-6ZGNRs transport model with 24 units in total with η = 0, 0.1, 0.5, 1.0 which is used to broaden the energy level in the leads to mimic metallic electrodes.

2. Band structures and PDOS of partially fluorinated F-6ZGNRs

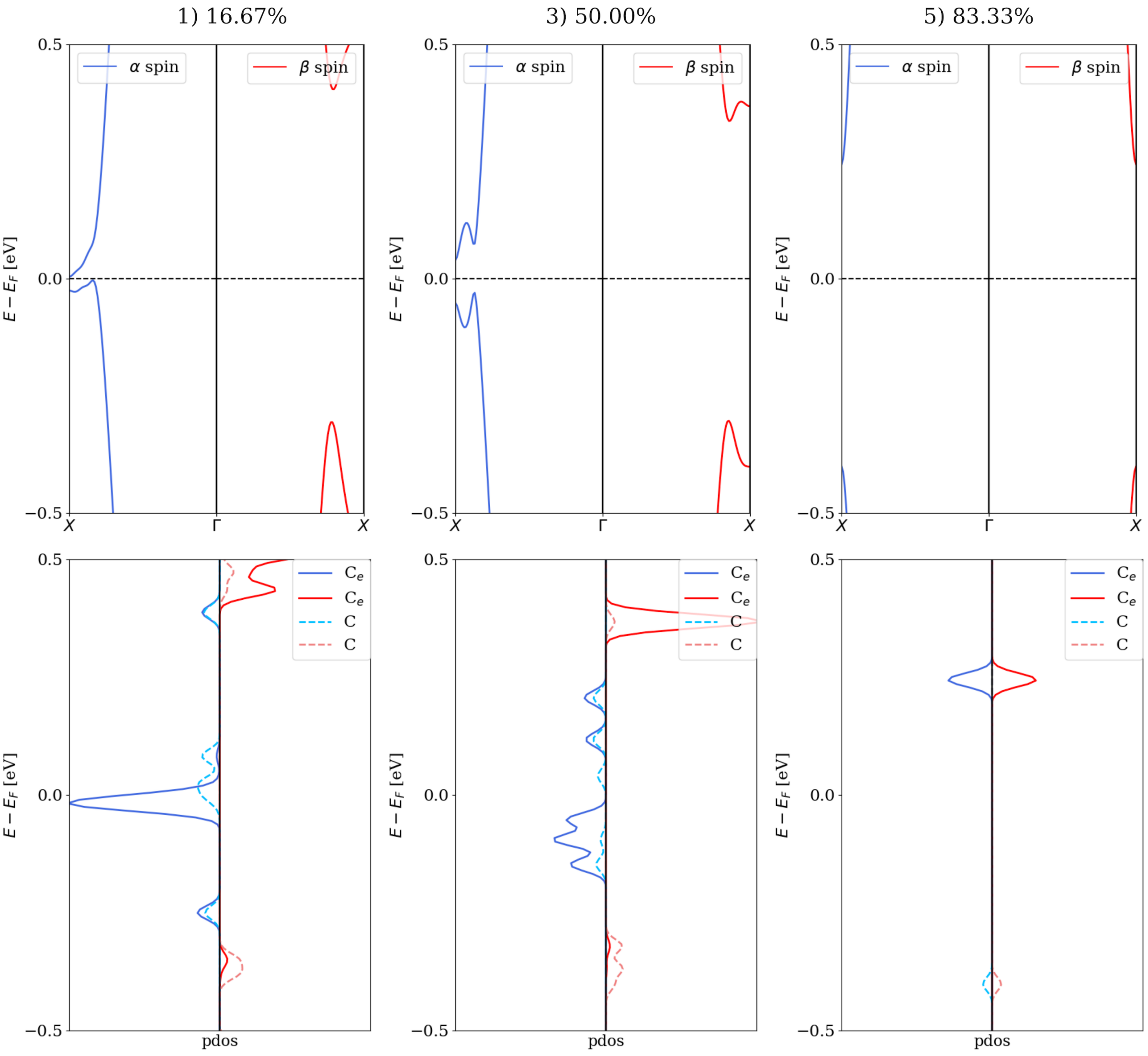

Figure S3: Bandstructures and PDOS of partially fluorinated F-6ZGNRs (C_e denotes edge ones, while C denotes the others). The structural models of the investigated systems are shown in Fig. 3 in the main manuscript. The α and β spin channels are presented in red and in blue, respectively.

3. Fluorinated F-8ZGNRs and F-12ZGNRs Structures

3.1 Fluorinated F-8ZGNRs structures

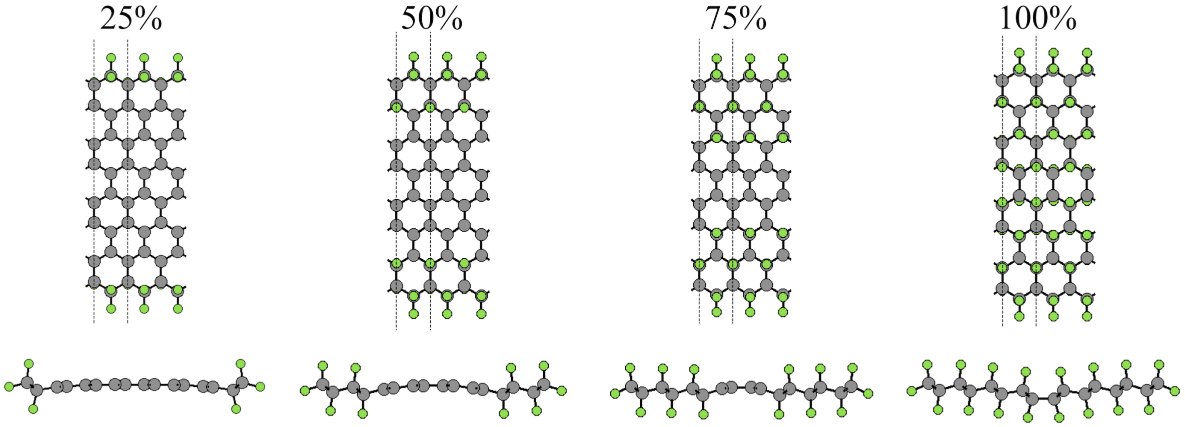

Figure S4: Top and side views of symmetric fluorinated F-8ZGNRs (PBE optimized structures) and the unit cell is marked with the dash lines in the top views. The xyz coordinates of the optimized structures will be available from the authors upon request.

3.2 Symmetrically fluorinated F-12ZGNRs structures

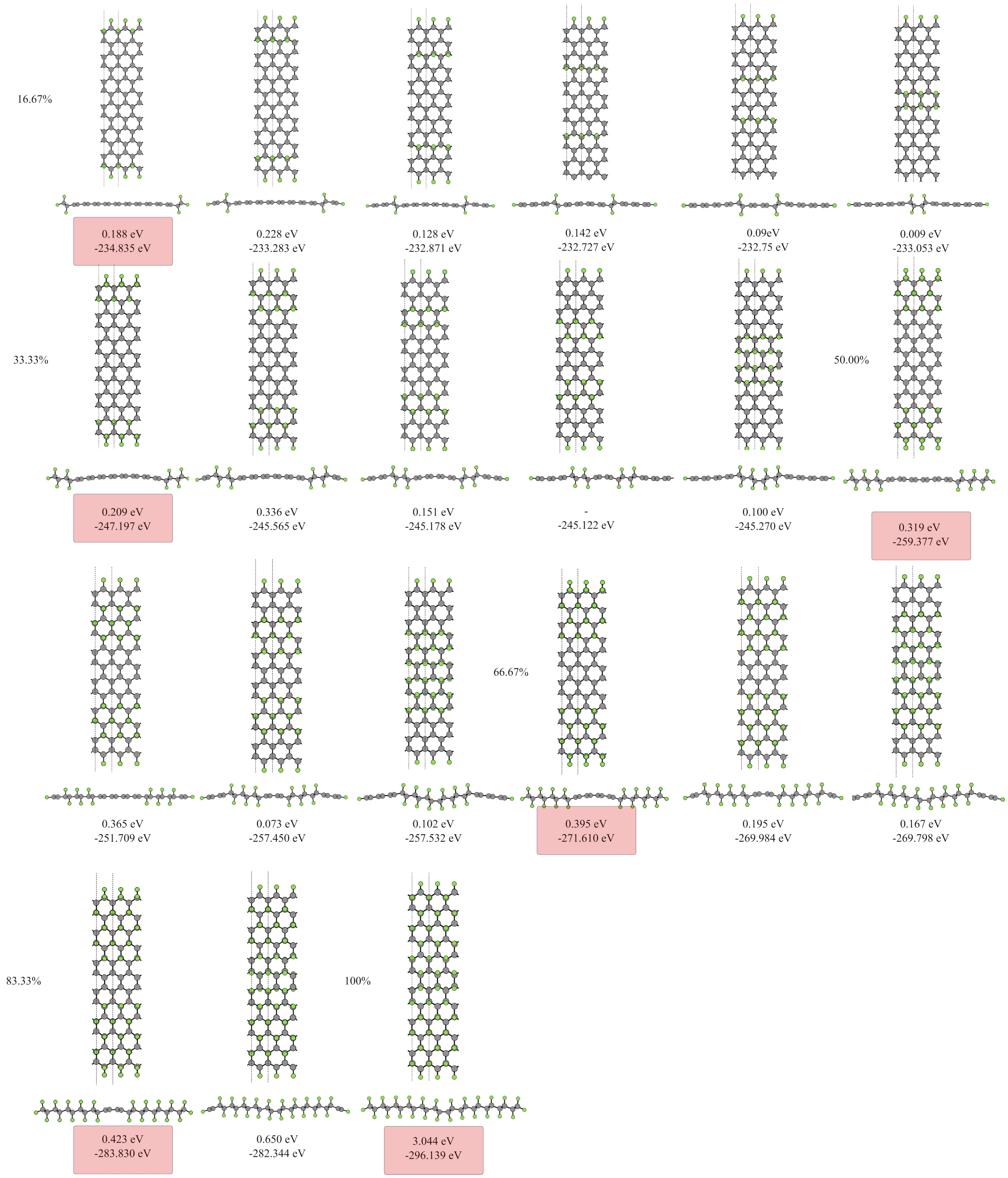

Figure S5: Top and sides views of symmetric fluorinated F-12ZGNRs (PBE optimized structures). The band gap values and the total energy are listed blow each structure. The structure within each fluorination degree obtained with the lowest energy is marked in red and is reported in the main text. The xyz coordinates of the optimized structures will be available from the authors upon request.

3.3 Symmetrically fluorinated F-12ZGNRs structures with Asymmetric Fluorine Orientation

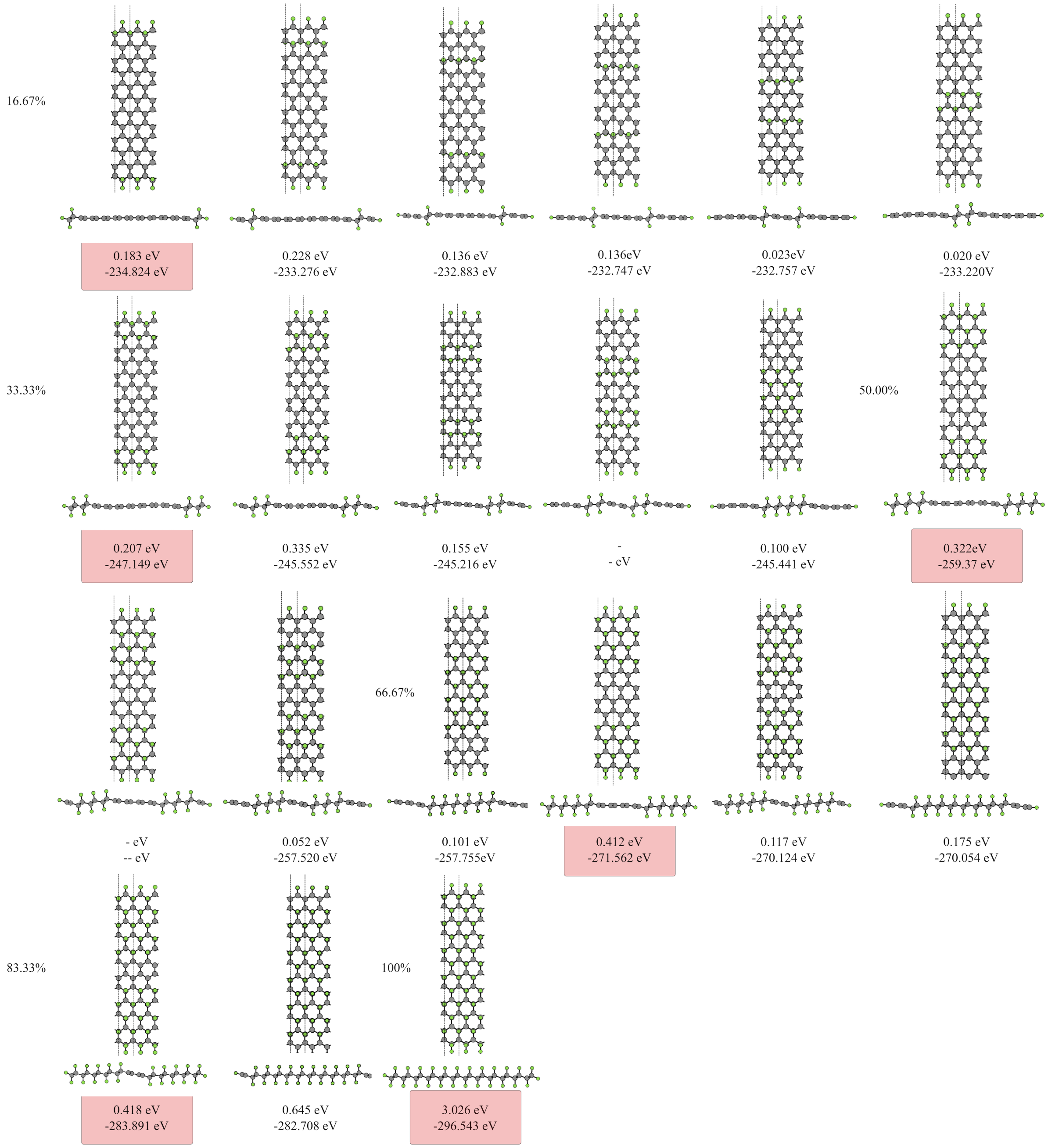

Figure S6: Top and sides views of symmetric fluorinated F-12ZGNRs with asymmetric fluorine orientation (PBE optimized structures). The band gap values and the total energy are listed blow each structure. The structure within each fluorination degree obtained with the lowest energy is marked in red. The xyz coordinates of the optimized structures will be available from the authors upon request.

4. Total current-bias voltage dependence of odd width of F-XZs

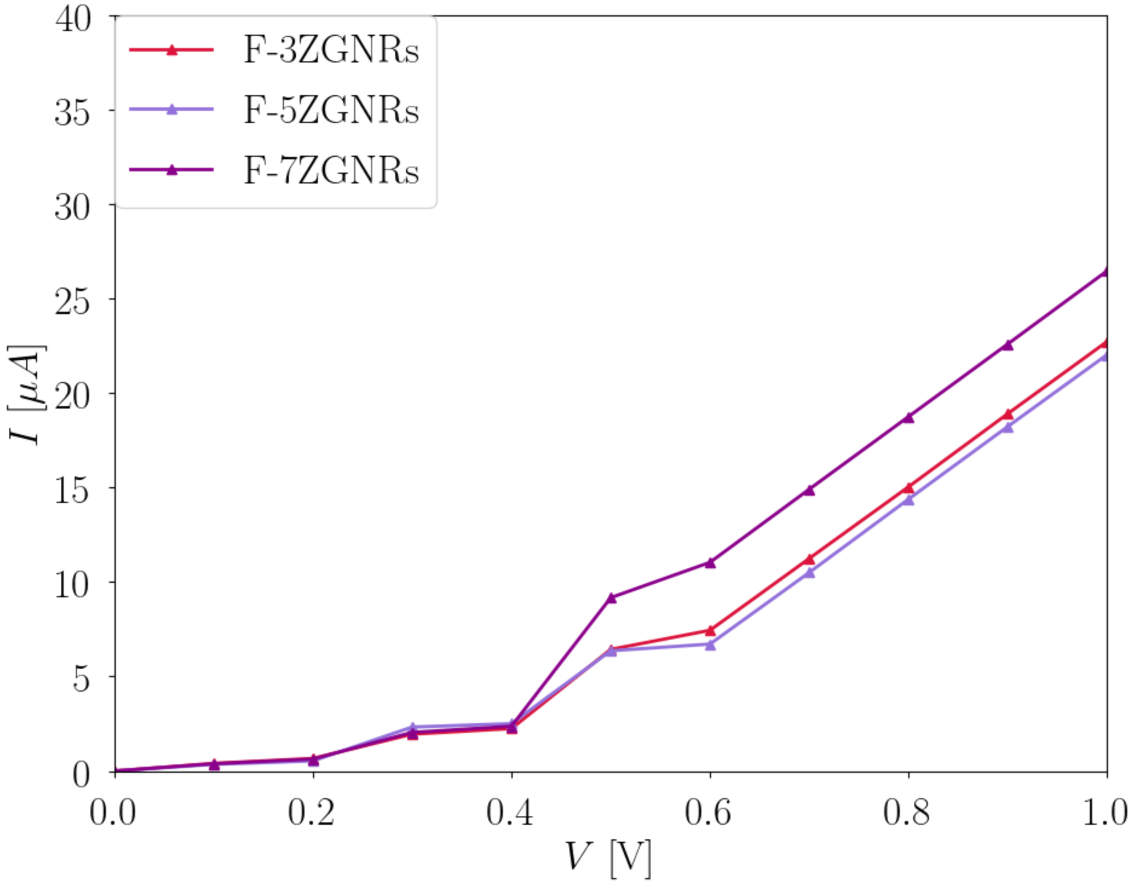

Figure S7: Total current I (μA) in dependence of the applied bias voltage V [V] with 0.1V step for the F-XZ transport model, where X = 3, 5 and 7.

5. Local Energy Spectra of Considered Structures

5.1 Local energy spectra of the scattering regions of considered even number width of F-XZs

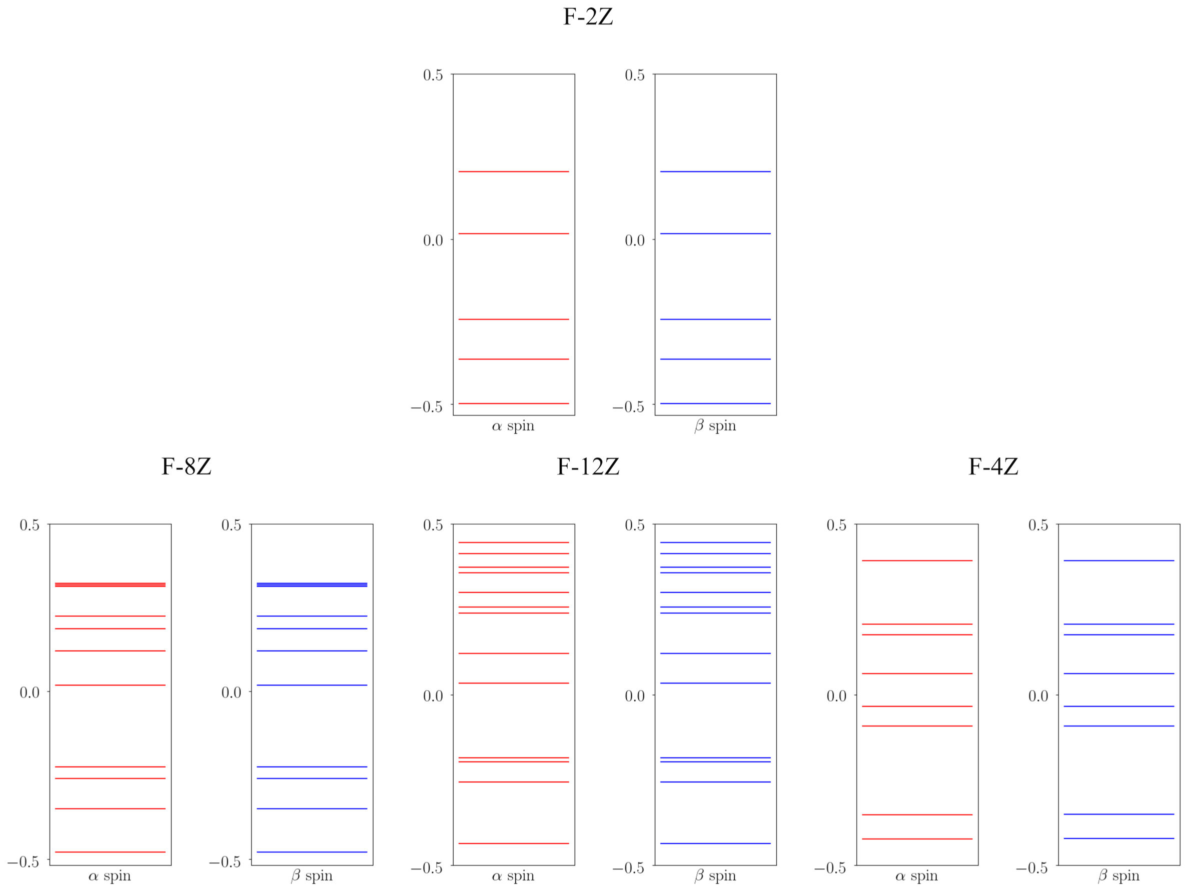

Figure S8: Local energy spectra of the scattering regions of considered even number width F-XZs, where X = 2, 4, 8 and 12.

5.2 Local energy spectra of the scattering regions of considered asymmetric fluorinated F-6Zs

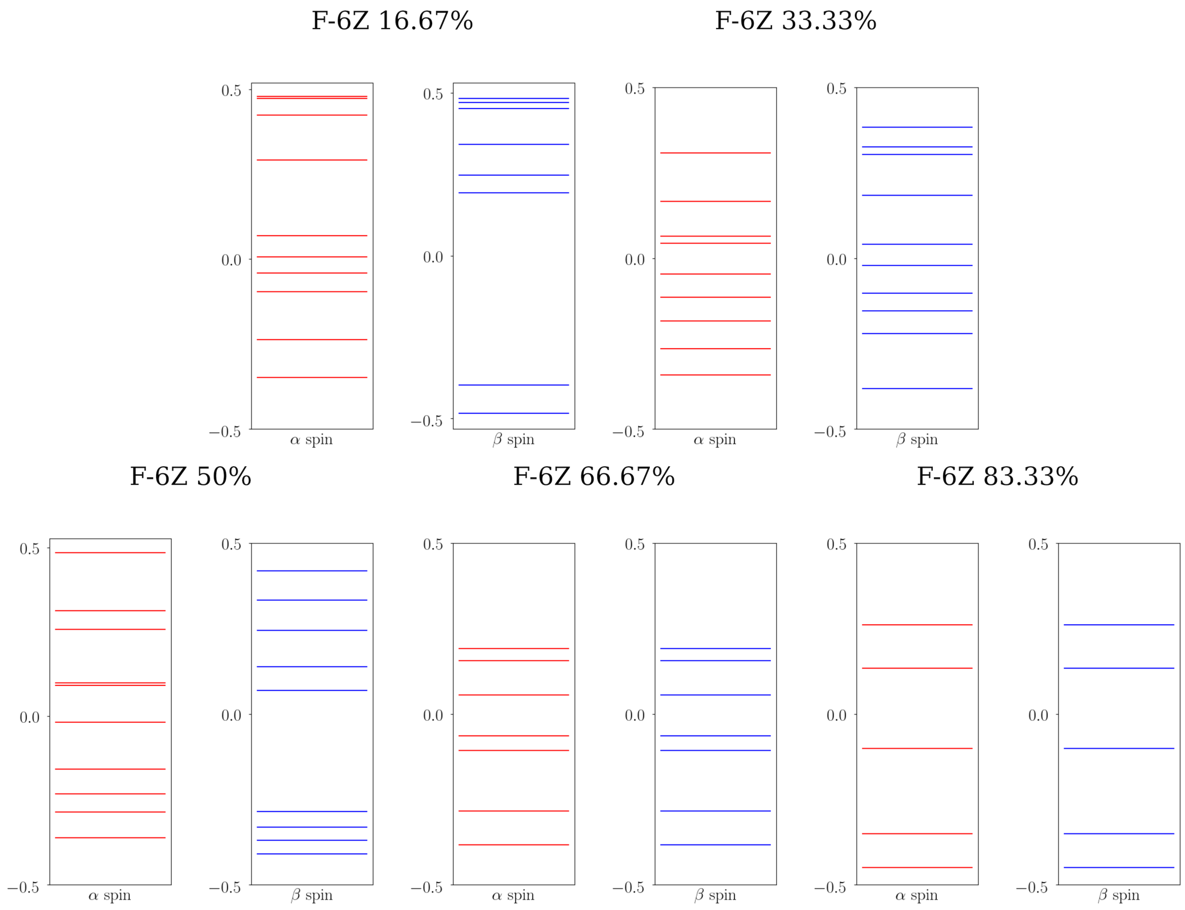

Figure S9: Local energy spectra of the scattering regions of asymmetric fluorinated F-6Zs.

5.3 Local energy spectra of the scattering regions of the symmetric fluorinated F-6Zs and F-8Zs

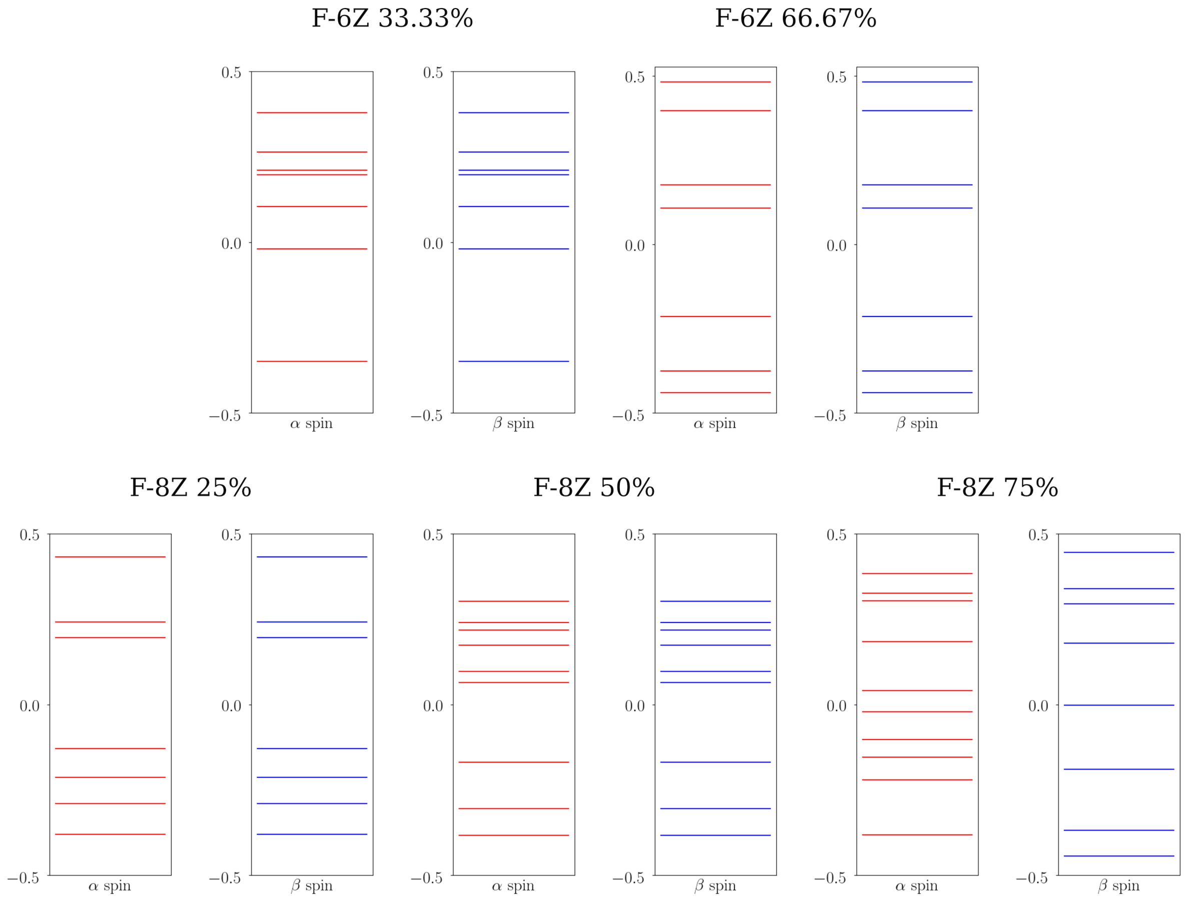

Figure S10: Local energy spectra of the scattering regions of the symmetric fluorinated F-6Zs and F-8Zs.
